# SOCIAL NETWORK AND MENTAL HEALTH OF OLDER CHINESE IMMIGRANTS IN AFFORDABLE SENIOR HOUSING DURING THE COVID-19 PANDEMIC

**DOI:** 10.1093/geroni/igac059.248

**Published:** 2022-12-20

**Authors:** Kexin Yu, Jiaming Liang, Yi-Hsuan Tung, Mutian Zhang, Luyan Yang, Shinyi Wu, Iris Chi

**Affiliations:** Oregon Health & Science University, Palo Alto, California, United States; University of Southern California, Los Angeles, California, United States; University of Southern California, Los Angeles, California, United States; University of Southern California, Los Angele, California, United States; University of Southern California, Los Angeles, California, United States; University of Southern California, Los Angeles, California, United States; University of Southern California, Los Angeles, California, United States

## Abstract

Chinese older immigrants who live in senior housing communities are at high risks of experiencing discrimination and social isolation during the COVID-19 pandemic. This study examines how and to what extent the pandemic has affected this population’s social network and mental health. Participants reported a decrease in social contact with their family and friends. Before the pandemic, many paid regular visits back to the home country and could not do so in the past two years. The loss of connection left some feeling despaired and expressed uncertainty on whether they could ever go back “home” before death. Participants also reported being in a low mood and feeling bored constantly. Participants reported resilience generated from their religious beliefs, having neighbors as role models, and wisdom learned from past life experiences. Knowledge produced in this project can inform the planning for responding to future crises in affordable senior housing.

